# Global regulatory reforms to promote equitable vaccine access in the next pandemic

**DOI:** 10.1371/journal.pgph.0002482

**Published:** 2023-10-18

**Authors:** Richard Mahoney, Peter J. Hotez, Maria Elena Bottazzi

**Affiliations:** 1 Texas Children’s Hospital Center for Vaccine Development, Department of Pediatrics, Division of Tropical Medicine, Baylor College of Medicine, Houston, Texas, United States of America; 2 Department of Molecular Virology and Microbiology, Baylor College of Medicine, Houston, Texas, United States of America; 3 Department of Biology, Baylor University, Waco, Texas, United States of America; 4 James A. Baker III Institute for Public Policy, Rice University, Houston, Texas, United States of America; 5 Hagler Institute for Advanced Study at Texas A&M University, College Station, Texas, United States of America; McGill University, CANADA

## Abstract

There is broad consensus that the global response to the Covid-19 pandemic was inadequate, leading to unacceptable levels of avoidable morbidity and mortality. Three strategic missteps led to the lack of equitable vaccine access: The heavy reliance on commercial vaccine manufacturers in high-income countries (HICs) versus low- and middle-income countries (LMICs); the emergence of vaccine nationalism restricting and delaying the supply of vaccines to LMICs; and an inadequate support or recognition for LMIC national regulatory authorities. To avoid these inequities in a future pandemic, we focus on three successful vaccine development and technology transfer case studies–the Hepatitis B vaccine produced in South Korea in the 1980s; the Meningitis A vaccine for Africa led by Program for Appropriate Technologies in Health (PATH) and the World Health Organization (WHO) in the 2000s; and a recombinant SARS CoV-2 protein-based vaccine technology from the Texas Children’s Hospital transferred to India and to Indonesia. In addition to expanding support for academic or non-profit product development partnerships, our analysis finds that an essential step is the strengthening of selected LMIC regulatory systems to become Stringent Regulatory Authorities (SRAs), together with a re-prioritization of the WHO Prequalification (PQ) system to ensure early vaccine availability in LMICs especially during pandemics. Advancing LMIC National Regulatory Authorities (NRAs) to Stringent Regulatory Authorities (SRAs) status will require substantial resources, but the benefits for future pandemic control and for health in LMIC would be immense. We call on the WHO, United Nation (UN) agencies and SRAs, to collaborate and implement a comprehensive roadmap to support LMIC regulators to achieve stringent status by 2030.

## Introduction

There is a broad consensus that the global response to the Covid-19 pandemic was inadequate and led to unacceptable levels of avoidable morbidity and mortality especially in lower- and middle-income countries (LMICs) [1].

This failure and pandemic response shortfalls had many, complex and not well understood causes. A leading cause was the rapid spread of the pandemic, which made it necessary, at an extraordinarily fast pace, to expand, improve and/or create global monitoring systems, health and economic impact analyses, new vaccines, diagnostics and other technologies, vaccine and diagnostics procurement mechanisms, supply-chain and global distribution strategies, flexible drug and vaccine regulatory systems, and public health disease control practices, as well as to implement related policies, systems and capabilities.

But perhaps the greatest shortfalls were associated with the supply of needed vaccines, diagnostics, and other essential technologies for people in LMICs [2]. One of the barriers to access of vaccines in developing countries was vaccine nationalism [3]. High-income countries (HICs), particularly the United States and member states of the European Union restricted the export of vaccines until they felt their own populations had received a sufficient supply. Even some LMICs such as India restricted exports [4]. Therefore, to address future pandemics more effectively, it will be necessary to have a different strategy for the research, development, manufacture, regulatory control, procurement, and distribution of vaccines.

## Vaccine development models in High-Income Countries (HICs)

The pandemic also led to some notable successes. In the United States, for instance, Operation Warp Speed (OWS), collaborations among the United States (US) Departments of Health and Human Services (HHS) and Defense (DOD), helped vaccine companies accelerate the development, testing and authorization of several new and effective vaccines for human use [1].

The OWS model consisted of providing very significant incentive funding to private pharmaceutical firms in HICs to develop and manufacture new COVID-19 vaccines. For example, the US Government was estimated to have spent $30 billion on its COVID-19 vaccine program, including approximately $25 billion for either the development or advanced purchase of mRNA vaccines from Pfizer and Moderna [5]. To induce the companies to participate in OWS, the US Government granted the companies specific ownership and/or control of related intellectual property rights (IPR) associated with their research and development (R&D) efforts, thus effectively creating company monopolies. These policies resulted in the companies obtaining extraordinary profits that enriched shareholders and company executives but did little to help people in LMICs [6].

There were many manufacturers in developing countries ready and willing to co-develop and produce the OWS-supported vaccines but were precluded by OWS and/or limited by HIC company policies [7]. Globally, the Coalition for Epidemic Preparedness and Innovations (CEPI) also played a role, contributing towards the acceleration of the development of vaccines and other biologic countermeasures against COVID-19, but early in the pandemic many of the manufacturers in developing countries were also not prioritized, focusing instead on support for HIC vaccine producers, including AstraZeneca, Inovio, Curevax, Novavax, Institut Pasteur, and others [8]. Another global effort, COVAX, the vaccine deployment pillar of the Access to COVID-19 Tools (ACT) Accelerator, was also created to help address the vaccine needs of LMICs, but it was unable to meet several key global targets and goals [9].

Most recently, Torreele et al. [10] have called for “transformational change” to ensure a more equitable situation for addressing the next pandemic and meeting the needs of LMICs. They state in part, “A transformative approach requires a fundamental change in why, how, where and by whom these technologies are developed and produced, and about who has access to this knowledge and know-how. The existing approach to R&D, manufacturing and access to and delivery of essential epidemic countermeasures is deeply inequitable, especially for people in LMICs, and for vulnerable populations worldwide.” They recommend the support of networked capabilities in LMICs that “have the guaranteed freedom to operate around technology platforms for vaccines…including with regard to IPR and technological know-how, and be financed adequately…”

We largely agree that expanding efforts to build capacity and to support vaccine research and development in LMICs is crucial, but also identify several additional key elements to ensure equitable vaccine access for future pandemics. The ultimate goal is to reduce the reliance of LMICs on HICs for technology to address their public health concerns.

To explain such factors. we discuss here three historical case studies and approaches to highlight how such change can successfully be implemented. First, the Hepatitis B vaccine developed by virologist Alfred Prince at the New York Blood Center (NYBC) and produced in South Korea in the 1980s; second, the Meningitis A vaccine for Africa led by Program for Appropriate Technologies in Health (PATH) and World Health Organization (WHO) in the 2000s; and third, the recombinant SARS CoV-2 protein-based vaccine technology from Texas Children’s Hospital Center for Vaccine Development at Baylor College of Medicine transferred to India and to Indonesia.

All these programs (see Table 1) emphasize how innovative technological and collaborative solutions with LMICs can overcome barriers erected by vaccine development models used in HICs and contribute significantly to improving global vaccine access.

**Table 1:** Innovative technological and collaborative case studies leading to vaccine global access.

Vaccine Case Study	Country Partnership	Technology Platform	Global Access Framework	Regulatory Framework
Hepatitis B Vaccine	US, South Korea	Hepatitis antigens derived from human plasma	World-wide exclusive license	WHO-Pre Qualification (PQ)
Meningococcus A Vaccine	US, India	Conjugate	No patent, Low cost	WHO-PQ
COVID-19 Vaccine	US, India, Indonesia	Recombinant Protein	Open-access, Non-exclusive licenses, No Patent, Low cost	India NRA, Indonesia

This paper addresses issues once a vaccine candidate has been identified and is ready for industrial manufacturing, clinical testing, authorization and distribution. Support for vaccine research especially in developing countries continues to be a crucial need, but that is not the focus of this paper. Instead, we find common ground in the need to improve regulatory support for LMIC national regulatory authorities (NRAs) and an urgency to afford them a status similar to those HIC NRAs identified as “stringent” by the WHO.

## Historical perspectives: From smallpox and rabies vaccines to Hepatitis B and Meningococcal A vaccines

The successes in technology transfer from Texas to LMICs build on several historical cases of a vaccine having been developed by a non-profit organization without patent protection and subsequently transferred to developing country manufactures for scale up, testing and distribution and with a goal of reaching the poor in LMICs.

The first case is the manufacture of smallpox vaccine originally developed by Jenner in 1796. For the smallpox eradication program of the 1960s and 1970s, a modified version of the original vaccine was manufactured by numerous facilities in LMICs, and they received extensive support and technical assistance from the Smallpox Eradication Program at WHO [11].

A second case is the development of rabies vaccines by Louis Pasteur in 1885 and the subsequent establishment of the Pasteur Institute in Paris with the objective of transferring its production technology to other Pasteur Institutes around the world [12].

In addition to the absence of any patent restrictions, a key reason why these two vaccines could be developed and distributed rapidly throughout the world was the absence, at the time of their invention, of regulatory frameworks managed by national governments that could review vaccine safety and efficacy and provide licenses for their production. WHO subsequently addressed the regulation of smallpox vaccine for the Eradication Program [11].

As regulatory requirements began to strengthen, first in the US and later in Europe and other developed countries from the 1950s on, fewer and fewer vaccine producers had access to the enormous resources necessary to build production facilities that met Good Manufacturing Practices (GMP) standards and to conduct the required development process especially clinical trials. In addition, countries struggled establishing and maintaining national regulatory systems.

Nevertheless, in modern times, there are two examples of transfer of vaccine production directly to developing country manufacturers. These are the development of a Hepatitis B vaccine by Alfred Prince and his laboratory at the NYBC and the work of the Meningitis Vaccine Project at PATH and WHO.

### The Hepatitis B vaccine

Dr. Alfred Prince was a leading figure in hepatitis B research and was responsible for identifying the hepatitis surface antigen as a marker of hepatitis B infection [13]. He surmised that a vaccine consisting of this antigen would be effective against hepatitis B virus infection. His laboratory developed a preparation method that consisted of “flash heating” plasma from people actively infected with hepatitis B virus. The flash heating killed the virus but left the hepatitis B surface antigen undamaged and immunogenic [14]. Prince was concerned about the high prices charged by commercial vaccine producer such as Merk and Merieux precluding use in LMICs. He wanted his vaccine to be cheap and available to the poor. In 1984, the New York Blood Center granted a world-wide exclusive license for manufacture of the vaccine to Cheil Sugar in South Korea. At this time, South Korea was very much a developing country with a per capita income of around $2500. In 1986, Prince with James Maynard and Richard Mahoney at PATH formed the International Task Force for Hepatitis B Immunization. With support from the James S. McDonnell Foundation, the Task Force succeeded in procuring the Cheil vaccine at $1 per dose and facilitated its introduction in several developing countries including Thailand [15]. A particular challenge in introducing the Cheil vaccine in developing countries was that it had regulatory approval only in South Korea. LMICs preferred that the vaccine be approved by a stringent regulatory authority (SRA) as designated by WHO or have the endorsement of WHO [16]. It was not financially feasible for Cheil to obtain regulatory approval by an Stringent Regulatory Agency (SRA) in the US or Europe. The developing countries that agreed to use the Cheil vaccine without stringent regulatory approval or the Strategic Advisory Group of Experts on Immunization (SAGE) endorsement did so on the basis that the use would be in highly controlled model immunization programs where surveillance for safety and efficacy (pharmacovigilance) would be rigorously conducted. The Task Force also worked with WHO staff to get hepatitis B vaccines on the SAGE agenda. SAGE endorsed hepatitis B immunization in 1992. It is noteworthy that although the NYBC had patents on this vaccine and licensed these patents to Cheil, the license did not prevent Cheil from offering the vaccine to LMICs at a very low price. The programs of the Task Force provided a platform on which later efforts by GAVI, the Vaccine Alliance, successfully introduced hepatitis B vaccine (as the tetravalent DPT-HBV) to many LMICs. In all, from the time of first development of Prince’s vaccine to wide availability in LMICs over two decades elapsed. This time lag could have been greatly shortened if the South Korean NRA had been assigned stringent status and was a designated SRA.

### The Meningococcal A vaccine

The Meningitis Vaccine Project (MVP) was an extraordinarily successful partnership between PATH and the WHO to develop, license, introduce and distribute conjugate meningococcal vaccines [17–19]. The project set a goal of a vaccine that would cost US $0.40 so that it could be affordable. After finding no European or U.S. manufacturers willing to make such a price commitment, MVP established a collaboration with the Serum Institute of India (SII), an LMIC vaccine producer in Pune, India. A safe and effective non-proprietary vaccine, i.e. no patents, with the desired price, was developed by SII and led to the almost total control of meningitis A in the meningitis belt of Africa. The success of this program was due in large part to its close association with WHO, which ensured that at every step of the way, the vaccine development and eventual manufacture met regulatory requirements including obtaining WHO Prequalification (PQ) [17]. Another key to the success of this program was a visionary ten-year funding commitment by the Bill & Melinda Gates Foundation, and the PATH leadership, including F. Marc LaForce. The MVP was a very successful initiative for a known infectious agent. From the completion of clinical trials of this vaccine to its availability for use in the Africa Meningitis Belt only a few years passed even though India did not have a SRA. This was largely because the regulatory barriers were addressed fully during the course of vaccine development.

It is impossible to know enough about a future pandemic infectious agent to launch a specifically targeted program similar to MVP or the Hepatitis B Task Force, but that was the original intent when CEPI was formed and launched various initiatives [20].

## A valuable COVID-19 vaccine development model–technological transfer from a hybrid academia- and hospital-based vaccine R&D center to an LMIC vaccine developer

Texas Children’s Hospital Center for Vaccine Development (Texas Children’s CVD) at Baylor College of Medicine successfully developed a COVID-19 vaccine technology that is now produced by two LMIC manufacturers [21].

This is the one of the most widely distributed COVID-19 vaccine technology developed, produced, and sold at low cost using a framework that specifically addressed, first and foremost, the public health needs in LMICs. We believe this program is an excellent illustration of the type of initiative recommended by Torreele [10].

This COVID-19 vaccine development framework was carried out by Texas Children’s CVD with Biological E in Hyderabad, India and, in parallel, with PT BioFarma in Bandung, Indonesia. These vaccine producers took the seed stocks for the production of the vaccine candidate (the SARS CoV-2 receptor binding domain (RBD)), which was engineered and developed in Texas and scaled up the manufacturing process for its commercial production while at the same time shepherding the vaccine evaluation through rigorous pre-clinical and clinical development stages and summarized in Hotez et.al., 2023 [21].

These recombinant RBD protein-based vaccines received regulatory approval by each country’s NRAs. The vaccines (CORBEVAX^TM^ in India available at ~US $3.00 per dose [22] and INDOVAC^TM^ in Indonesia), were produced using a well-established technology: a yeast-based expression system for the production of recombinant proteins such as the platform used for the production of the world’s most widely used hepatitis B vaccine [21].

There are several elements that distinguish this vaccine development framework program used by Texas Children’s CVD: 1) their prior coronavirus (Severe Acute Respiratory Syndrome (SARS) and Middle Eastern respiratory syndrome (MERS)) vaccine R&D experience; 2) the comprehensive regulatory-enabling R&D steps followed, which led to a smooth but robust technology transfer to the manufacturers; 3) the deliberate selection of the vaccine technology platform focusing on microbial fermentation in yeast with a track record of producing the largest number of doses, leading to highly safe, effective, and low cost vaccines and, 4) the decision not to patent or protect the engineered COVID-19 vaccine technology, which enabled an easier transfer directly to the developing country manufacturers contributing towards the goal to co-develop a COVID-19 vaccine that would meet the needs of the poor not only in India and Indonesia but with the potential to extend its reach in other LMICs [21].

Since 2011, Texas Children’s CVD had been developing and testing recombinant protein vaccines comprised of the receptor binding domain of coronaviruses that cause both SARS and MERS [21]. These vaccine prototypes elicited high levels of both virus neutralizing antibodies and cellular immune responses and were protective in virus challenge models [23–25]. When the COVID-19 genome was made available in January 2020, Texas Children’s CVD coronavirus vaccine program pivoted to this new virus, and a prototype vaccine was developed and successfully tested including in a non-human primate virus challenge model [26]. Each step in the vaccine development process was published in the open access biomedical literature available on the PubMed National Library of Medicine database [27–31]. Baylor College of Medicine then non-exclusively licensed the vaccine technology on favorable terms and without patent protection to several LMIC vaccine producers.

Amongst these, as mentioned above, two producers, one based in India (Biological E) and in Indonesia (BioFarma) advanced rapidly the scale-up and testing of their vaccines rapidly. Both were selected on the basis of mutual interest and due diligence in terms of past track records and successes in producing yeast-based recombinant vaccines at large scale, with acceptable quality and affordable prices. Ultimately, these actions led to the production and delivery of CORBEVAX^TM^ for India and INDOVAC^TM^ the halal-certified vaccine for Indonesia, even though neither vaccine programs received OWS support and overall, minimal support from CEPI and/or the group of 7 (G7) nations or their financial instruments.

The Texas-based COVID-19 vaccine program linked to developing country vaccine producers, provide a proof-of-concept that it is possible in the modern age to produce safe and effective vaccines for pandemic threats even in the absence of major G7 support or the involvement of large pharmaceutical firms based in the US or Europe. However, it was also limited in its impact to a great extent by regulatory difficulties. For example, in the case of CORBEVAX^TM^, while the vaccine was approved by the Drug Controller of India, the national regulatory of authority of India is not considered a SRA. Therefore, in order for other international agencies and LMICs to purchase the vaccine, the manufacturer had to initiate a process to obtain WHO PQ; (PQ is a WHO program involving an assessment process used by the United Nations (UN) and other procurement agencies to make decisions about procuring specific products. It involves assessment of both production facilities and NRAs). More than one year after CORBEVAX^TM^ emergency authorization in India and when it first went into pediatric arms, this vaccine has not yet received WHO PQ [32]. A similar situation is happening with INDOVAC^TM^ in Indonesia [33].

Since their approvals, many other LMICs expressed interest in authorizing and acquiring CORBEVAX^TM^ or INDOVAC^TM^. However, it is likely that the absence of WHO PQ for these 2 vaccines created a regulatory barrier for LMICs to acquire and import these vaccines. Despite these challenges, these two vaccines have been amply administered in India and Indonesia. Currently, CORBEVAX^TM^ is continues to be used as a booster vaccine in individuals previously vaccinated with COVAXIN^TM^ and COVISHIELD^TM^, and INDOVAC^TM^ continues to be used as a booster vaccine in individuals previously vaccinated with Pfizer mRNA vaccines.

The Texas Children’s CVD vaccine technology continues to be a critical tool in the race to outpace new variants of COVID-19. Ahead of the May 18, 2023, WHO COVID Vaccine Composition Advisory Group announcement, which was followed by the June 15, 2023, FDA’s Vaccine and Related Biological Products Advisory Committee (VRBPAC), Texas Children’s CVD was already working towards the development of a new monovalent (single strain) vaccine prototype against the XBB strain [34, 35]. A new monovalent COVID-19 vaccine is an ideal strategic option for supporting the global immunization infrastructure. The omicron specific (XBB) recombinant protein booster is in development by Biological E but the path for WHO PQ remains uncertain for this vaccine as well.

## The global regulatory framework

The continuing evolution and increasing sophistication of regulatory regimes for assessment of the safety and efficacy of vaccines and for assessing the capability of production facilities to produce vaccines that meet accepted standards has resulted in a bifurcated world.

WHO has recently initiated a new framework for evaluating and publicly designating regulatory authorities as WHO Listed Authorities (WLA) [36]. This initiative seeks “to develop a transparent and evidence-based pathway for regulatory authorities operating at an advanced level of performance to be globally recognized, thereby replacing the procurement-oriented concept of stringent regulatory authorities. Of the 56 countries included in the WHO Listed Authorities (WLAs) in 2022 to have SRA designation, none are in LMICs [37]

Obtaining PQ from WHO is a complicated, time-consuming, and costly process. The partial list of the steps a manufacturer must take [38, 39] are shown in Table 2. Once these steps are completed, WHO may inform relevant UN agencies that the vaccine is suitable for procurement.

**Table 2:** Partial list of steps a manufacturer takes to obtain WHO-PQ.

Manufacturer Initial Submission Steps	Submission after WHO Programmatic Suitability of Vaccine Candidates for WHO Prequalification Standing Committee Review
Show the vaccine meets mandatory characteristics for programmatic suitability as defined by WHO	Submit vaccine samples for testing by WHO-contracted laboratories
Obtain marketing authorization from its NRA	Undergo a site audit conducted by WHO experts (may include recommendations for improvements at the NRA)
Submit a Product Summary File including: • Personnel details • premises and equipment • vaccine composition, presentations and schedules • production, quality control and stability • clinical experience • production and distribution data • update on regulatory actions	
An application letter providing details of country and sites of manufacture, licensing status and the presentations put forward to United Nations agencies for procurement	
Demonstrate the vaccine is in compliance with the mandatory programmatic characteristics as defined by WHO	

## Discussion

The Prince Hepatitis B vaccine, the PATH/WHO Meningitis A vaccine, and the COVID vaccine technology developed at Texas Children’s CVD with LMIC manufacturers provide important lessons for the development of future vaccines including for pandemic infections that would prioritize manufacture and delivery meeting the needs of the populations in LMICs.

The models lead to the identification of the following key elements:

1. Implementation by product development partnerships led by either an academic-based (e.g., Texas Children’s CVD) or a non-profit organization (e.g., PATH, NYBC) that delivers expertise and resources.
2. Technology transfer directly to developing country vaccine manufacturers in a non-proprietary manner that allows the licensee to offer the vaccine at a very low price affordable by LMICs.
3. In parallel or in advance of elements 1 & 2, the need for the strengthening of national and international regulatory systems to accelerate approval of the vaccine for human use both in the country of manufacture and in other developing countries.

In all three cases, the most significant rate-limiting step was the type of national regulatory framework available for each manufacturer and the hurdles of obtaining PQ from WHO because India, Indonesia and South Korea did not have SRAs. In the case of the Meningitis A vaccine, this barrier was addressed by including WHO in the program. HIC manufacturers, on the other hand, do not have to contend with PQ because their NRAs are SRAs. WHO procedures allow for PQ to be granted automatically to producers in countries with SRAs [39].

**Fig 1:**
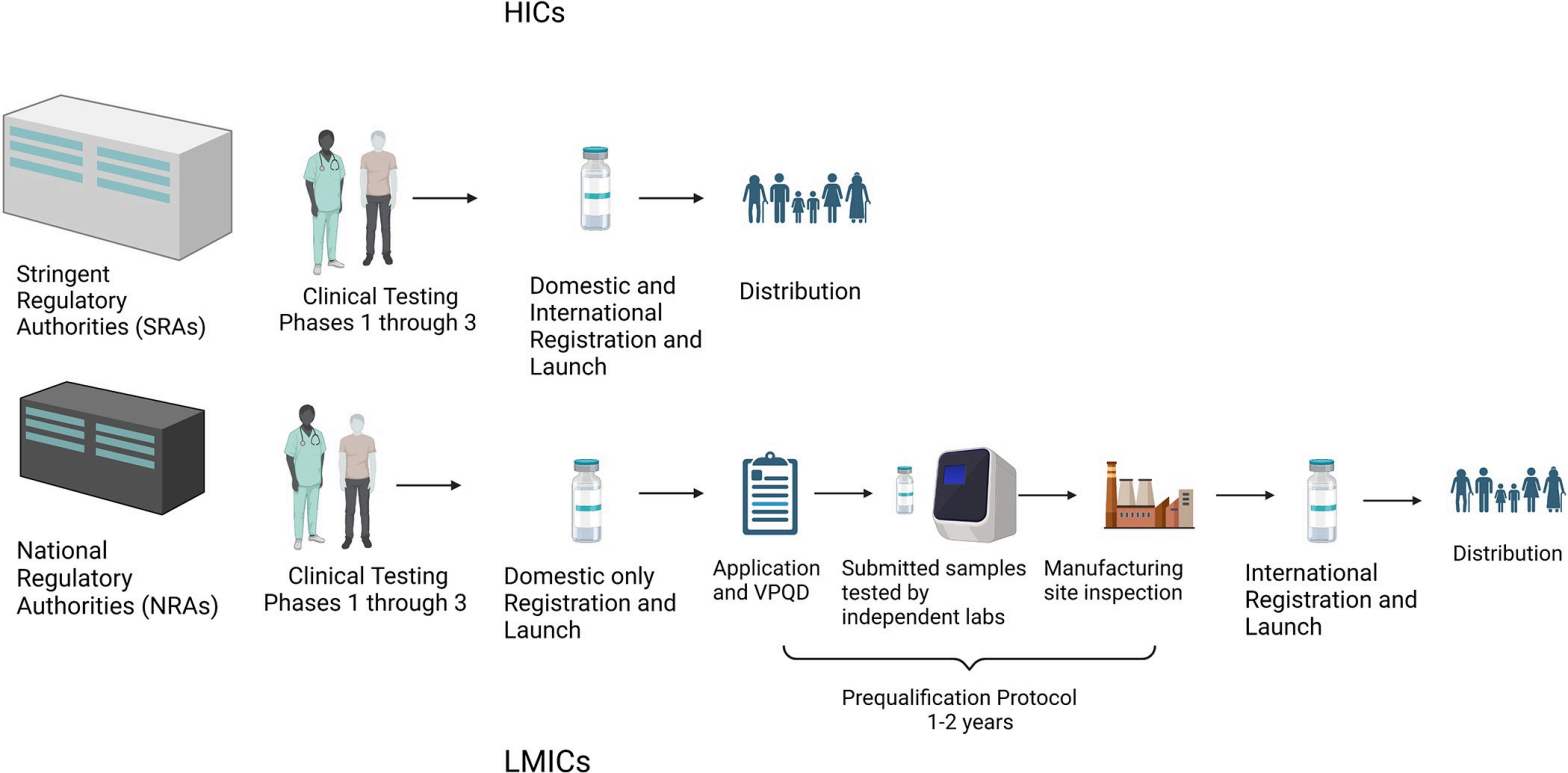
Vaccine development process for High-Income Countries (HICs) versus Low- and Middle-Income Countries (LMICs). Created with BioRender.com.

The diagram illustrates (Fig 1) the vaccine development process including the additional regulatory hurdles faced by LMIC producers needed to receive both domestic and international approval of their vaccines compared to producers in countries with SRAs. LMIC producers are capable of undertaking all activities at the same speed as HIC producers, except they have to obtain PQ which results in extensive delays in undertaking export. If this barrier is not addressed by implementing the recommendation made here, it is likely that LMICs will face the same delay in obtaining vaccine supplies in future pandemics as they did with the Covid-19 pandemic.

Thus, we recommend that high priority should be accorded to a two-pronged approach: 1) Recognizing the importance of academic or non-profit product development partnerships in promoting innovation to produce vaccines in collaboration with LMIC vaccine producers, and 2) improving NRAs in vaccine producing LMICs to the level of SRAs so that their products may be expeditiously available for international distribution. We recommend steps to expedite the work of LMIC vaccine producers and prioritize their efforts to produce and distribute safe and effective low-cost vaccines. These would include both vaccines for pandemic threats and neglected tropical diseases. The urgency for G7 or G20 nations to support capacity building for these vaccine producers has been detailed previously in reports from a Lancet Commission on COVID-19 [40] and other convening entities. Here, however, we emphasize the prioritization for improving NRAs in vaccine producing LMICs to the level of SRAs so that their products may be expeditiously available for international distribution. This will require participation of not only the LMIC vaccine producers and their NRAs and other government entities, but also strategic and focused assistance from NRAs currently designated as stringent, together with the WHO and possibly other UN agencies and CEPI, and GAVI, the Vaccine Alliance.

In both 2012 and 2020, the National Academy of Medicine (and its forerunner the Institute of Medicine) looked at ongoing efforts to strengthen food and drug regulatory systems abroad [41, 42]. This detailed assessment and report provides a set of recommendations for further action by WHO, donors, financial institutions, and U.S. agencies (NIH, FDA, USAID) to dedicate resources to helping improve the performance of NRAs and LMIC manufacturers. In addition, it calls on national governments to provide greater support including capacity building to NRAs.

Moreover, an excellent and comprehensive framework for enhancing the regulatory system has been proposed by McGoldrick, et al. [41]. PAHO is leading the Pan American Network for Drug Regulatory Harmonization [43]. The African Medicines Regulatory Harmonization (AMRH) program has been working since 2009 to increase cooperation among regulatory agencies on the continent [44].

However, these initiatives are primarily concerned with harmonization of regulatory systems among countries so as to reduce the burden of making multiple and varying submissions to a large number (~100) of regulatory agencies. While this is a valuable goal, we believe that an additional and essential step is the upgrading of a select number of NRAs in vaccine producing LMICs to SRA status.

We therefore call on the WHO and other UN agencies, as well as the current SRAs to issue a comprehensive roadmap for advancing several LMIC SRAs to achieve stringent status by 2030. This could include the designation of at least one SRA each on the African Continent (the newly formed African Medicines Agency (AMA) is being formed for this purpose) [45] in South Asia, and in the Latin American and Caribbean region.

It is interesting to consider what would have happened with the COVID-19 pandemic, if India, Indonesia, and several other LMICs had SRAs. Licensure in those countries would have immediately allowed for export to all other countries in the world, especially LMICs. For instance, Biological E has the capacity to produce at least one hundred million of doses per month of a billion doses per year of CORBEVAX^TM^. The prices would have been much less than those paid by COVAX and, in many cases, the countries themselves.

In addition to initiatives to help LMICs establish SRAs, an interim step could be to forge regional initiatives in which NRAs would agree to work together to review and approve new vaccines. This strategy promises to greatly reduce the time and cost of obtaining regulatory approval in multiple developing countries. This approach was being successfully implemented for the Sanofi dengue vaccine by the Dengue Vaccine Initiative (DVI) of the International Vaccine Institute in coordination with WHO [46]. This approach was not fully implemented following the difficulties encountered with the Sanofi vaccine in the Philippines and Brazil. The AMA will potentially help meet this need.

Another option that is being seriously considered in Africa is the establishment of new or expanded production facilities in LMICs. While this approach could overcome the problem of vaccine nationalism, at least for the African countries where this new production capacity is created, it would face major hurdles including reaching the economies of scale currently achieved by many LMIC producers such as the Serum Institute of India [47].

## Conclusions

The vaccine development strategy of the COVID-19 pandemic in which vast resources were allocated mostly to HIC producers to take over and finalize the development of vaccines that had initially been developed largely in academia and government was a failure in terms of meeting the needs of people in LMICs. Vaccine nationalism and the inertia of the global health enterprise greatly impeded LMIC access to needed vaccines. Therefore, a major overhaul of the global pandemic response ecosystem such as proposed by Torreele [10] will be necessary.

Ensuring that people in LMICs can receive vaccines rapidly after the outbreak of a pandemic will depend in large part on the availability of vaccine producers in LMICs. The greatest barrier to LMIC producers being able to supply other LMICs is the necessity to meet regulatory requirements and, especially, to obtain WHO PQ. The highest priority should be capacity building and strengthening support to selected LMIC NRAs to help them become SRAs. In the meantime, additional assistance should be provided to LMIC manufacturers so they can expeditiously obtain PQ. WHO has recognized this need and is prepared to provide technical assistance to LMIC manufacturers [48]. However, this program has limited funding.

A second barrier is the lack of recognition for academic-based and non-profit product development partnerships as innovators that can work hand-in-glove with LMIC vaccine producers. The three examples here provide essential case studies.

Pandemic vaccine development considerations could be seen as the justification for a broad program to enhance LMIC NRAs to SRAs [49]. Implementing such a program would be a necessary step for LMICs to achieve greater self-sufficiency in developing, approving, supplying and monitoring needed vaccines, drugs and devices for their populations and especially for the diseases that are important in their countries. In turn, to maintain SRA status within an LMIC, would require considerable resources and time and should be seen as necessary for improving health in LMICs in addition to ensuring a more effective response to future pandemics [50].

Furthermore, the existence of SRAs in vaccine producing LMICs and, in the meantime, of a robust rapidly acting WHO PQ system that gives priority to LMIC producers will facilitate and encourage vaccine developers, especially those in academia or the non-profit world, to prioritize the transfer of their vaccine to LMIC producers.

Our recommendations address one of the major issues that impeded an effective response to the COVID-19 pandemic, the urgency to strengthen regulatory systems in LMICs to become SRAs, together with a re-prioritization of the WHO Prequalification (PQ) system to ensure rapid actions for LMICs especially during pandemics. To address these hurdles, we call on the WHO, UN agencies and SRAs, to collaborate and implement a comprehensive roadmap to support LMIC regulators, including the AMA, to achieve stringent status by 2030. The designation of LMIC-based SRAs will empower local vaccine innovations and facilitate vaccine development with regionally appropriate frameworks leading to accessible vaccines.
